# Round Table: Discussion with families and lay associations

**DOI:** 10.1186/1750-1172-10-S2-O28

**Published:** 2015-11-11

**Authors:** Tiziana Mongini, Alessandra Gambineri

**Affiliations:** 1 Neuromuscular Center, Department of Neurosciences ‘Rita Levi Montalcini’, University of Turin, Turin, Italy; 2 Division of Endocrinology, Department of Medical and Surgical Science (DIMEC), S. Orsola-Malpighi Hospital, Bologna, Italy

## 

This session was dedicated to patients, their relatives and delegates of Family Associations (the Progeria Family Circle, the European Association of Progeria Families; AIProSaB, the Italian Association for Progeria, Sammy Basso; AIDMED, the Italian Association for Emery-Dreifuss Muscular Dystrophy; the Associazione Alessandra Proietti for Emery-Dreifuss Muscular Dystrophy), with the main objective to establish for the first time a direct interaction with the scientific international community working on laminopathies. Since the patient number is very low, and the clinical presentations of laminopathies vary greatly among the different subgroups, this was a good opportunity to confront the whole community to identify the ‘unmet needs’; besides the therapy to cure the disease, they include all the complementary aspects of the disease that worsen the patient quality of life. With the help of patients, the researchers may plan adequate strategies to address all the issues related to the disease and to give a ‘priority’ to each of them. Some examples of such positive interactions are the primary role of Patients Association in the production of the Standards of Care for a group of neuromuscular disorders by TREAT-NMD, an European Organization, the involvement of Parent Project in DMD clinical trials planning by Pharma Industries and their inclusion in the process of drug approval by American and European Drug Agencies (FDA and EMA). In Italy, the Italian Association of Myology produced two consensus conferences, on vaccinations and on anesthesia procedures in neuromuscular patients, on the suggestion of Italian Neuromuscular Associations.

With the exception of patients with Progeria, who already benefit from a good network of expert that cover their needs, the other patients, in particular those with muscular dystrophy, are less characterized and their clinical protocols are less standardized.

Future possible areas of common interests, to be prioritized and supported by Patients Association, may include a consensus on precise criteria to define the different phenotypes; protocols to reduce diagnostic delay or misdiagnoses; physiotherapy indications; pain relevance and management; nutritional issues.

Patient participation during the round table allowed us to focus on their main issues: the need to spread knowledge on laminopathies and to foster research activity in the field (mentioned by an EDMD parent and by an HGPS patient); the need of clear indications for follow-up reference centers (mentioned by an EDMD patient); concerns about dietary advices for muscular and progeroid laminopathies (mentioned by an EDMD parent with reference to the talk by Dr. Quijano-Roy and comments by Dr. D'Amico and by an HGPS parent).

